# Automatic Coronary Wall and Atherosclerotic Plaque Segmentation from 3D Coronary CT Angiography

**DOI:** 10.1038/s41598-018-37168-4

**Published:** 2019-01-10

**Authors:** Ahmed M. Ghanem, Ahmed H. Hamimi, Jatin R. Matta, Aaron Carass, Reham M. Elgarf, Ahmed M. Gharib, Khaled Z. Abd-Elmoniem

**Affiliations:** 1 The Biomedical and Metabolic Imaging Branch, National Institute of Diabetes, Digestive, and Kidney Diseases, National Institutes of Health, Bethesda, MD USA; 2 The Image Analysis and Communications Laboratory, Department of Electrical and Computer Engineering, Johns Hopkins University, Baltimore, MD USA

## Abstract

Coronary plaque burden measured by coronary computerized tomography angiography (CCTA), independent of stenosis, is a significant independent predictor of coronary heart disease (CHD) events and mortality. Hence, it is essential to develop comprehensive CCTA plaque quantification beyond existing subjective plaque volume or stenosis scoring methods. The purpose of this study is to develop a framework for automated 3D segmentation of CCTA vessel wall and quantification of atherosclerotic plaque, independent of the amount of stenosis, along with overcoming challenges caused by poor contrast, motion artifacts, severe stenosis, and degradation of image quality. Vesselness, region growing, and two sequential level sets are employed for segmenting the inner and outer wall to prevent artifact-defective segmentation. Lumen and vessel boundaries are joined to create the coronary wall. Curved multiplanar reformation is used to straighten the segmented lumen and wall using lumen centerline. *In-vivo* evaluation included CCTA stenotic and non-stenotic plaques from 41 asymptomatic subjects with 122 plaques of different characteristics against the individual and consensus of expert readers. Results demonstrate that the framework segmentation performed robustly by providing a reliable working platform for accelerated, objective, and reproducible atherosclerotic plaque characterization beyond subjective assessment of stenosis; can be potentially applicable for monitoring response to therapy.

## Introduction

Coronary heart disease (CHD) is the leading cause of death around the world^1^. The build-up of atherosclerotic plaques often results in severe coronary artery lumen stenosis which, in conjunction with plaque rupture, is the main etiology of angina, myocardial infarction, and sudden death^2,3^. The severity of the plaque build-up, also known as plaque burden, is multi-faceted involving the evaluation of coronary artery plaque content, volume, distribution, and lumen stenosis. While stenosis directly affects the flow of blood in the coronary arties, the spreading and composition of plaque may have a more abrupt and detrimental role in the development of CHD^2,4^. Small to moderate sized soft lipid-rich plaques can be more harmful than larger hard or calcified ones because of their increased vulnerability to rupture resulting in thrombosis and sudden disruption of blood flow^2,3^. Additionally, due to the positive arterial remodeling^5^ in the early stages of plaque development, the change in lumen cross-sectional area may not be perceived by imaging and hence, concealing the actual size and vulnerability of plaques. In fact, plaques that are vulnerable to rupture are typically lipid-filled non-stenotic and exist at many locations along the arteries^6^. Indeed, nearly half of sudden cardiac deaths occur in otherwise asymptomatic patients from culprit plaques that are not flow-limiting^7^. Therefore, measuring the degree of stenosis only is insufficient for complete assessment of the risk of CHD, particularly in the asymptomatic stages of the disease.

Assessment of plaque burden based on lumen stenosis scoring was driven by the availability of lumen-only data from conventional X-ray coronary angiography. With the advances in CCTA, 3D data now provide more in-depth insight about all components of plaque burden; the CCTA measurements have shown to be more precise independent predictors of CHD than conventional risk metrics^8,9^. However, identifying plaque locations and sizes from CCTA is still a tedious manual task that is prone to subjective image interpretation. Hence, a more objective and user-agnostic method is desirable for measuring and documenting the extent of coronary plaque burden as identified by CCTA.

### Background and Related Work

Many paradigms were introduced for coronary artery lumen segmentation and stenosis detection from CCTA images. Optical flow techniques use visual cues (such as local changes in the lumen inner diameter) to guide segmentation, whereas machine learning approaches attempt to learn features that may not be recognizable or perceivable to human experts. Examples of optical flow approaches include the use of a Corkscrew tracking-based vessel extraction technique^10,11^ for lumen segmentation and centerline calculation. Marquering *et al*.^12^ employed a fast marching level set to estimate the initial lumen contour and a model-guided minimum cost approach^13^ to find the final contour. Wang *et al*.^14^ started with an initial centerline and iteratively applied level set and distance transformations to estimate the final centerline and vessel border. Schaap *et al*.^15^ employed the intensities along a given centerline to guide a graph cut algorithm for lumen segmentation. These techniques were limited to specific plaque types, e.g., calcified plaques. They were also only demonstrated in a limited number of cases. The performance of these techniques was generally sensitive to the initial centerline accuracy, the length of the stenosis, and the artery cross section shape.

Machine learning-based techniques have been used to detect stenosis directly or segment lumen first then calculate the stenosis. Zuluaga *et al*.^16,17^ assumed the lesion is a local outlier compared to normal artery regions and employed intensity-based features with use of Support Vector Machines (SVM) to detect such outliers. The proposed metric was calculated in planes orthogonal to a given centerline, thus the final accuracy is also influenced by the given initial centerline. Further details about related coronary artery segmentation and stenosis detection techniques can be found in Kirisli *et al*.^18^.

Other approaches detect stenosis by first detecting plaques. Kitamura *et al*.^19^ proposed a multi-label graph cut technique based on higher-order potentials and Hessian analysis to detect stenosis that exceeded 20%. Kang *et al*.^20^ proposed a two-stage technique. In the first stage, two independent stenosis detectors were applied: an SVM and a formula-based analytic method. In the second stage, an SVM based decision fusion algorithm used the output of the two detectors to provide a more accurate detection for lesions with stenosis of greater than 25%. Sivalingam *et al*.^21^ proposed a hybrid segmentation technique to segment the vessel wall. This technique used active contour models and a random forest regression with the segmentation being evaluated on five arteries with calcified plaques, mixed, or both.

In addition to the focus on the detection of lumen stenosis, the majority of current techniques only detect stenosis that exceeds 20%. This presents little or no information about performance with small to moderate soft plaques that are an important contributor to CHD and predictor of future events, as noted earlier.

In this work, we propose the first framework for 3D coronary CTA wall and plaque segmentation, regardless to the degree of stenosis, with a particular interest in the soft plaques of all sizes that cause mild or insignificant lumen stenosis. We compare its performance in a cohort of asymptomatic CHD subjects against three-expert individual and consensus delineations of the outer and inner lumen wall in different plaque size categories.

### Problem Definition

Unlike coronary lumen segmentation, which only focuses on delineating the high-intensity lumen at the inner wall border from the low-intensity wall tissue and surroundings, the segmentation of coronary artery wall and plaques from CCTA images is substantially more challenging. Current CTA systems exist that provide relatively high temporal resolution around 66 msec., and a spatial resolution around 0.33 × 0.3 × 0.3 mm^3^, which are reasonable for the assessment of cardiac function and significant coronary artery stenosis. However, the available resolution adds restrictions to the accurate measurement of the vessel wall thickness, which typically ranges from 0.75 ± 0.17 mm for healthy segments (segments without plaques) to 4.38 ± 0.71 mm for segments with stenosis ≥40%^22^. Moreover, the current temporal resolution is not sufficient to eliminate all motion artifacts. Contrast also presents an issue between the vessel outer wall and surrounding tissues being substantially low; particularly when the vessel is very close to or surrounded by the myocardium and lacking the surrounding adipose tissue. These many challenges increase the complexity of segmenting the vessel wall and small plaques and therefore mandate the utilization of a multi-discipline framework.

### Strategy Outline

**Figure 1:**
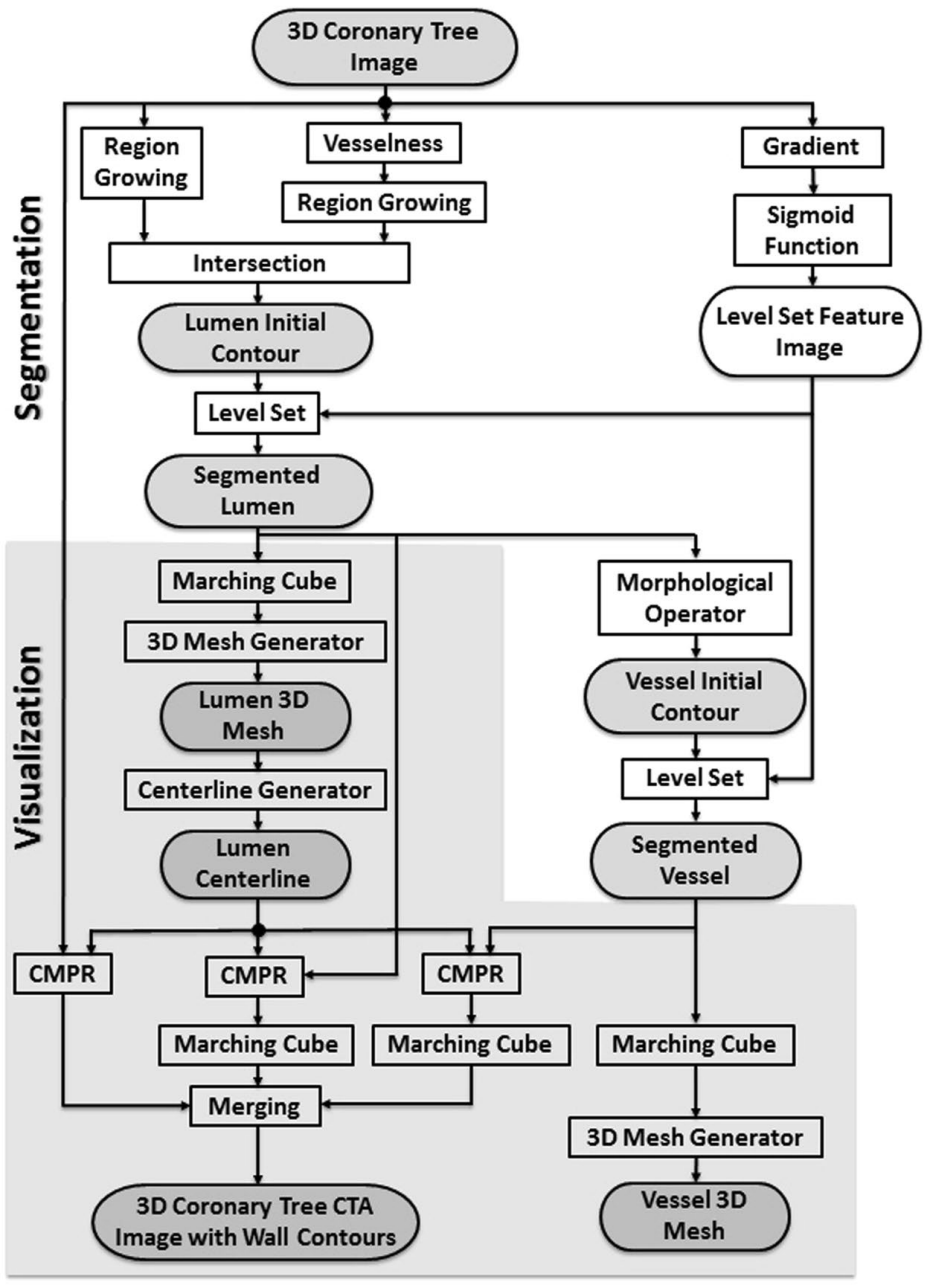
Lumen inner vessel boundary and outer vessel boundary segmentation and visualization block diagram.

The proposed framework is split into two major parts: segmentation of the coronary wall and visualization of the output. Segmentation has four modules as shown in Fig. 1. These modules are: S1) Lumen “inner wall” initial contour using Hessian analysis and region growing; S2) Vessel “outer wall” initial contour using mathematical morphology; S3) Final lumen segmentation; and S4) Final vessel segmentation, both using level sets. Visualization includes: V1) Generating a lumen 3D mesh; V2) Generating a vessel 3D mesh, both using the marching cube methods; V3) Lumen centerline using computational geometry; and V4) Curved multi-planar reformation (CMPR).

The proposed framework will be described in the subsequent Methods and Materials section.

## Results

### Framework experiments and evaluation

The experiments were part of a study that was approved by the local institutional review board (IRB) at the National Institutes of Health, in compliance with the Declaration of Helsinki, and were performed in accordance with relevant guidelines and regulations. Forty-one asymptomatic CAD subjects (18 females) were included in the performance assessment after signing informed consent. Participants were recruited using local advertisement from year 2011 to year 2016 (ClinicalTrials.gov identifier: NCT01399385). The criteria for inclusion were: age ≥20 years, without obesity, diabetes mellitus, or known history of cardiovascular disease.

#### Error and reproducibility assessment

Two observer radiologists, each with over 15 years of experience in coronary CTA imaging and interpretation, were involved in the study as investigators and to assist in the reading of the data and the evaluation of the framework performance. CCTA images were acquired using a 64-slice multi-detector CT (MDCT) or higher using previously established techniques^23–25^. Agatston coronary calcium score (CaS) was obtained without contrast^26^, then a standard coronary CCTA was performed to obtain images of the coronary vessels using iodine contrast.

#### Coronary CTA plaque localization and characterization

Before applying the proposed segmentation framework, CCTA images were read by one of the radiologist co-authors to identify the segments of adequate diagnostic image quality and the extent of atherosclerosis plaque burden therein. Axial, multi-planar reformatted coronary images were obtained using a 3-dimentional software tool (Virtual Place; AZE, Tokyo, Japan)^24^. Additionally, both trans-axial and reformatted images were used, and a modified 16-segment model was used by the radiologists to visually identify coronary segments^8,27^. In each of the 16 coronary segments, plaques were scored by the radiologist for their overall characteristics including: plaque presence (0 = No, 1 = Yes), plaque type (0 = None, 1 = Soft, 2 = Mixed, 3 = Calcified), plaque volume (0 = None, 1 = Small/trace “> = 1 to < = 24%”, 2 = Mild “> = 25 to< = 49%”, 3 = Moderate “> = 50 to < = 74%”, 4 = Large “> = 75%”) relative to the segment length^28,29^, and plaque luminal stenosis severity (0 = None: 0% luminal stenosis, 1 = Mild: 1% to 49% luminal stenosis, 2 = Moderate: 50% to 69% luminal stenosis, or 3 = Severe: 70% luminal stenosis)^9^. Based on the radiologist visual interpretation and assessment, a report for each subject was created listing the location, type, size of each plaque, and the degree of stenosis caused by the plaque. Inclusion in the study stopped when at least 20 plaques of each volume category were obtained.

**Figure 2:**
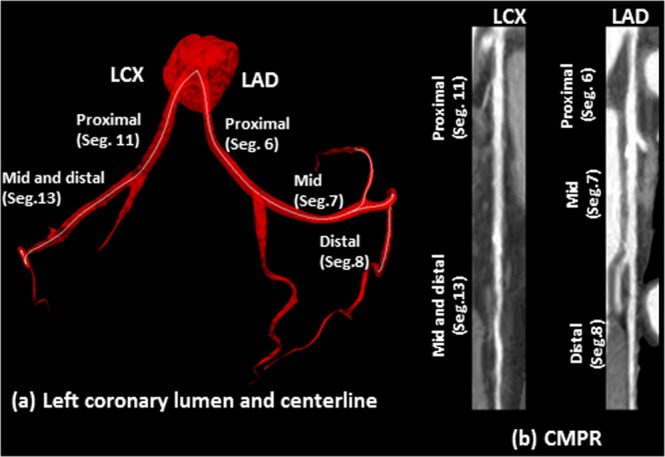
An example of the centerline of LCX and LAD (**a**), which is generated following the segmentation of the inner and outer walls. The centerline is used to generate the straightened arteries using CMPR (**b**).

The framework execution begins when the radiologist first identifies the origins of the right coronary artery (RCA) and the left main coronary artery (LM) by selecting two points at their ostia. The coronary tree lumen and wall were then automatically extracted according to the framework in Fig. 1. Accordingly, lumen and vessel boundaries were delineated, centerlines were generated, and the vessels were straightened and presented to the radiologist as in Fig. 2. Next, the radiologist was asked to identify the proximal and lateral ends of each of the previously recorded plaques. Plaque wall 3D meshes, locations, and lengths were added to the records. Segments with inadequate diagnostic image quality or with vessel caliber less than 2.0 mm, exclusive of focal stenosis, were excluded from the assessment. Further detailed step-by-step routines and software library calls be found in the attached appendix.

#### Comparison with Coronary CTA Consensus reading

Twenty-five arteries were chosen sequentially from 19 subjects containing seventy plaques including at least 15 plaques of each size (small, mild, moderate, large). Each 3D straightened artery was presented to the three observers as 5 longitudinal sections radially concentric at the centerline of the artery and at an angular gap of 36°. The observers were then asked individually to delineate the lumen and vessel contours unaware of the delineation results from the framework. A week later, the delineated plaques were randomly presented to observers together and a consensus reading was obtained, which will be considered the reference segmentation.

The blinded manual delineation of each observer as well as the automatic framework delineation were individually compared to the reference segmentation. Comparisons included the three extracted volumes; lumen, vessel, and wall, using the following similarity and error metrics.

Similarity:

1 $$DICE({V}_{t},{V}_{r})=\frac{2\cdot {\sum }_{i=1}^{n}({V}_{{t}_{i}}{\cap }^{}{V}_{{r}_{i}})}{{\sum }_{i=1}^{n}({V}_{{t}_{i}}+{V}_{{r}_{i}})}\times 100 \% $$

Volume Mean Squared Error:

2 $$VolMSE({V}_{t},{V}_{r})=\,\frac{1}{n}\sum _{i=1}^{n}{({V}_{{t}_{i}}-{V}_{{r}_{i}})}^{2},{\rm{and}}$$

Relative Volume Error:

3 $$({V}_{t},{V}_{r})=\frac{{\sum }_{i=1}^{n}(|{V}_{{t}_{i}}-{V}_{{r}_{i}}|)}{{\sum }_{i=1}^{n}(|{V}_{{r}_{i}}|)}\times 100{\rm{ \% }}.$$

Here, ${V}_{t}$ is the set of test plaque volumes, which in this experiment is either one of the observers or the automatic framework volume. The set ${V}_{r}$ is the reference plaque volume set, which in this experiment is the volume obtained by consensus reading, a*n*d *n* is the number of plaques.

#### Significance of discrepancy between framework and observer

In many situations, the difference of delineation between the algorithm and the radiologist is either under the noise floor level or in regions of large uncertainty and therefore there is no superiority of one delineation over another for adequate representation of a plaque. The goal of this experiment is to quantify the unacceptable segmentation error in our framework. That is the error which represents a clear deviation from the true edges and requires correction by a radiologist. A larger sample size of 122 plaques sliced at equally-spaced 20 longitudinal radially concentric sections at the centerline of the artery. The first radiologist reader was asked to manually adjust the framework-generated contours if needed.

The performance criteria in Eqs (1–3) were assessed as well as precision and sensitivity defined by

4 $$PREC({V}_{t},{V}_{r})=\frac{{\sum }_{i=1}^{n}({V}_{{t}_{i}}{\cap }^{}{V}_{{r}_{i}})}{{\sum }_{i=1}^{n}{V}_{{t}_{i}}}\times 100 \% ,$$

and

5 $$SENS({V}_{t},{V}_{r})=\,\frac{{\sum }_{i=1}^{n}({V}_{{t}_{i}}{\cap }^{}{V}_{{r}_{i}})}{{\sum }_{i=1}^{n}{V}_{{r}_{i}}}\times 100 \% ,{\rm{respectively}}{\rm{.}}$$

Here, ${V}_{t}$ is the test volume, which in this experiment is the automatic framework volume. ${V}_{r}$ is the reference volume, which in this experiment is the volume obtained by the expert radiologist after correcting the framework-generated volume.

### Framework execution

**Figure 3:**
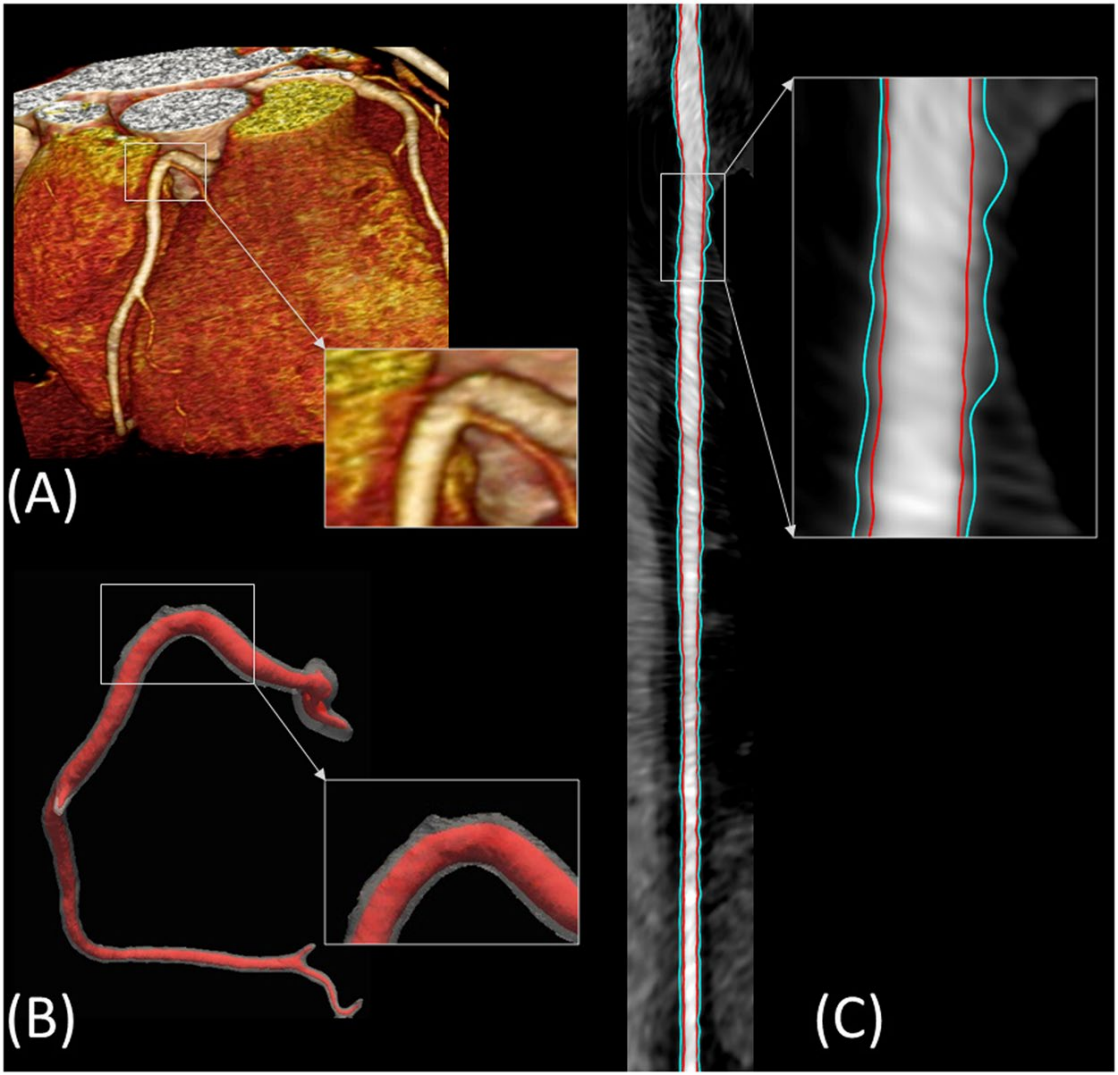
Visualization of framework segmentation. (**A**) The input 3D coronary CTA dataset. (**B**) Segmented lumen (red) and wall (gray). (**C**) Straightened vessel demonstrating the delineated inner and outer boundaries of the vessel in red and blue, respectively.

**Figure 4:**
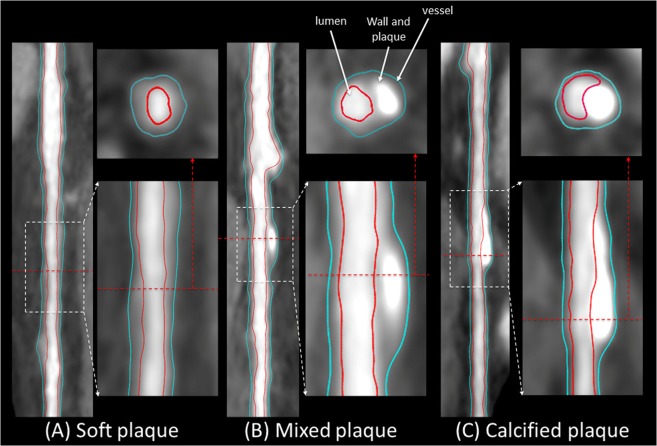
Reformatted coronary artery with (**A**) soft plaque, (**B**) mixed plaque, and (**C**) calcified plaque. The framework-segmented lumen inner contours and vessel outer contours are overlaid in red and blue, respectively.

A typical execution of the framework is shown in Fig. 3 in which the original 3D coronary CTA is fed to the framework, lumen and wall are then segmented. The lumen centerline is calculated and used to straighten the segmented lumen and wall as well as the original gray-level 3D image. The radiologist was able to visualize the different concentric longitudinal and parallel transverse cross-sections as shown in Fig. 4. The typical execution time of the proposed framework is 56 seconds per artery while our expert radiologists took in average 9 minutes per artery, not including CMPR, to manually set the centerline and segment the lumen and vessel outer boundaries for five longitudinal sections.

#### *In Vivo* error and reproducibility assessment

Participants’ characteristics were the following: median age = 52 y.o. with the interquartile range (IQR) (43.6–58.0), median body mass index (BMI) = 25.26 (IQR: 23.34–28.44), median Framingham score = 2.75 (IQR: 0.30–7.42), and median Atherosclerotic Cardiovascular Disease (ASCVD) score = 3.67 (IQR: 0.90–6.30). 122 atherosclerotic plaques were identified. These included 95 soft plaques, 20 mixed, and 7 calcified. Based on size, 36 plaques were small, 34 mild, 30 medium, and 22 large. Based on degree of stenosis, plaques were categorized into 45 without stenosis, 58 mild, 13 moderate, and 6 severe.

Table 1 summarizes the performance of the three observers and framework segmenting the three coronary vessel volumes (lumen, vessel, and wall) relative to the consensus delineation. Automatic framework volumes were always superior to at least one observer. When compared to the three observers, the framework automatic segmentations ranked first with the least volume MSE, the errors were 8.0 mm^3^, 20.9 mm^3^, and 18.4 mm^3^ for the lumen, vessel, and wall, respectively. In the relative volume error, the framework ranked third in the lumen, second in the vessel, and first in vessel wall segmentation with relative errors of 26.1%, 21.5%, and 25.4%, respectively. Finally, for the DICE metric, the framework ranked third in lumen and wall, and ranked second in vessel segmentation with DICE coefficient of 81.1%, 60.2%, and 81.1%, respectively.

**Table 1:** Performance of the three observers and the framework in delineating the lumen, the vessel, and the wall. Criteria included volume and similarity deviation from the consensus volumes and shapes.

	Lumen (inner boundaries)			Vessel (outer boundaries)			Vessel Wall		
	Volume MSE (mm^3^)	Rel. Volume Error (%)	Similarity (%)	Volume MSE (mm^3^)	Rel. Volume Error (%)	Similarity (%)	Volume MSE (mm^3^)	Rel. Volume Error (%)	Similarity (%)
Observer 1	9.8	20.8	84.9	26.0	16.8	86.4	25.1	26.3	67.5
Observer 2	21.1	42.9	68.3	40.6	31.1	79.5	28.2	31.4	51.6
Observer 3	13.0	22.6	82.9	49.8	39.1	79.4	42.8	53.1	63.3
Framework	8.0	26.1	81.1	20.9	21.5	81.1	18.4	25.4	60.2
Rank	1st	3rd	3rd	1st	2nd	2nd	1st	1st	3rd

**Figure 5:**
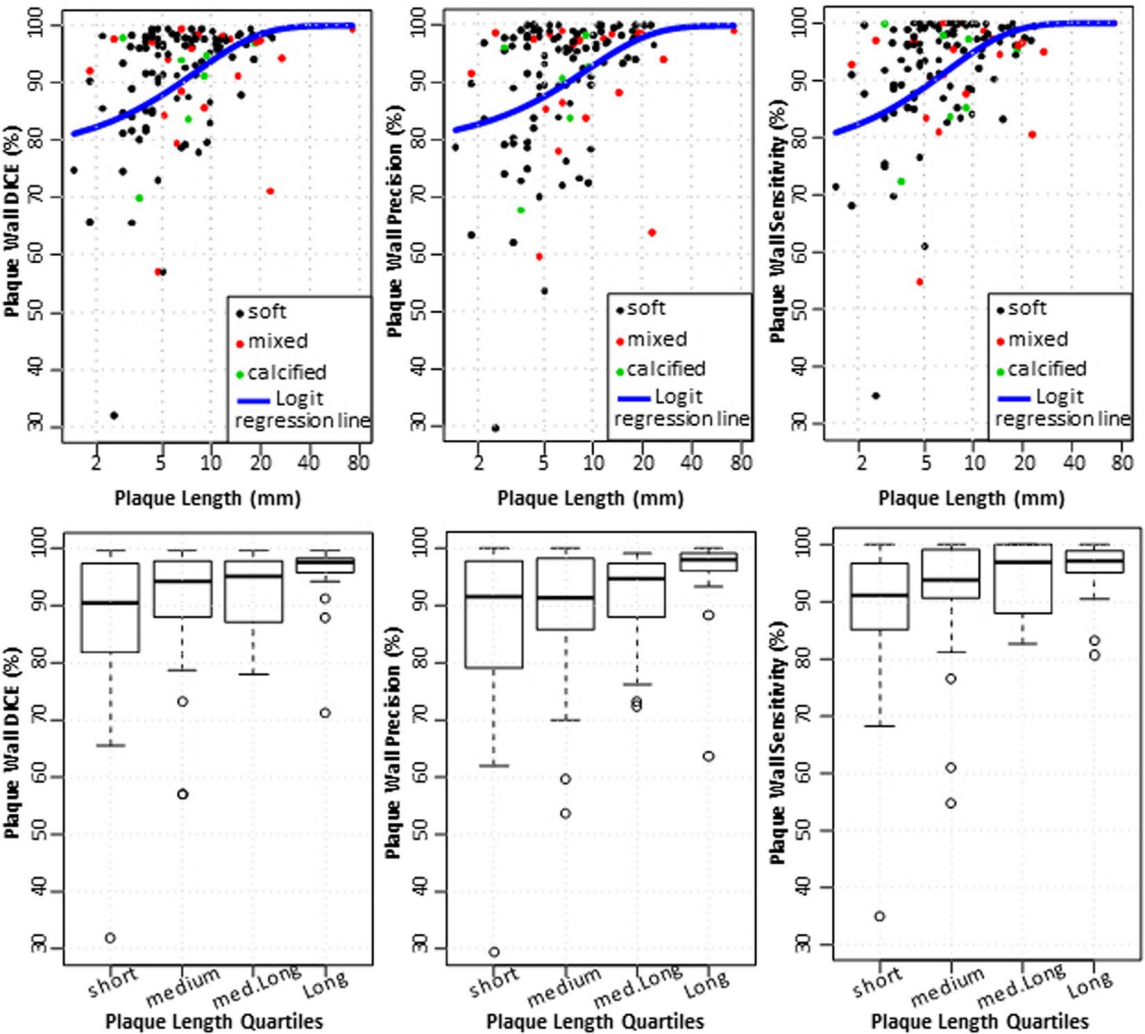
DICE, precision, and sensitivity performance metrics for wall delineation using algorithm compared to expert observer manual segmentation. Results are plotted vs. plaque length in mm (top row), and vs. plaque length quartiles (bottom row).

**Figure 6:**
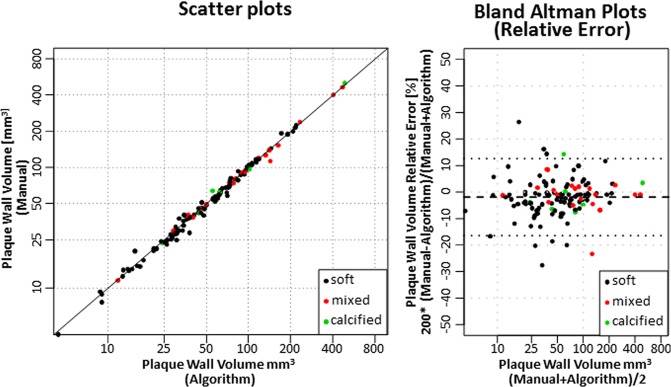
Scatter plots and Bland-Altman graphs for the coronary plaque volume delineation using the framework vs. expert manual segmentation.

The results of the second experiment that involved framework segmentation versus manual supervised segmentation are demonstrated in this section. For plaque DICE, measurements are presented in Fig. 5. In the small plaques, median DICE, PREC, and SENS scores were 90%, 92%, and 90%, respectively. The quality measures improved to above 95% for large plaques. The interquartile ranges (IQR) also narrowed substantially to near 5% as plaques get larger. Lumen and vessel DICE, PREC, and PREC trends were similar to those of plaque wall albeit with higher median (above 95%) and narrower IQR that starts around 6% and narrows to as small as 3% (results not shown). Framework and expert segmentations were highly correlated without substantial deviation. Actual and relative plaque volume errors were within ±10 mm^3^ and 12%, respectively (Fig. 6).

## Discussion

Coronary artery disease (CAD) starts and continues to accumulate deposits of plaques silently for decades before the development of the symptoms. During this asymptomatic phase, plaques grow mainly outwards or with minimal stenosis. Many recent large clinical studies demonstrated that in asymptomatic subjects, plaque burden including number, size, in addition to the luminal encroachment of the plaques, is a strong independent predictor of future CAD events and mortality^8,9,28^.

While there are multiple studies that addressed the problem of coronary lumen stenosis detection, there are few that attempts to systematically delineate coronary wall from CTA, particularly for asymptomatic CAD subjects. In this study, we developed and implemented a framework for automatic segmentation of coronary artery wall from CCTA in asymptomatic subjects at low and intermediate Framingham risk profile. Yet, there is evidence that they develop a substantial amount of plaque burden and even outward positive remodeling that cause no stenosis but may well rupture and become life-threatening. This group is of particular interest for a preventative approach towards early detection and quantification of CAD thereby improving disease monitoring and treatment outcome. This is the stage at which intervention could be more successful and cost-effective.

CCTA often produces an inconsistent and shallow gradient of contrast between the coronary wall and surrounding tissues. Additionally, the thin coronary wall is surrounded by a background with a wide range of attenuations. Collectively, these present multiple unique challenges toward robust delineation of plaques and walls and therefore mandate the development of novel multi-algorithm processing approaches.

**Figure 7:**
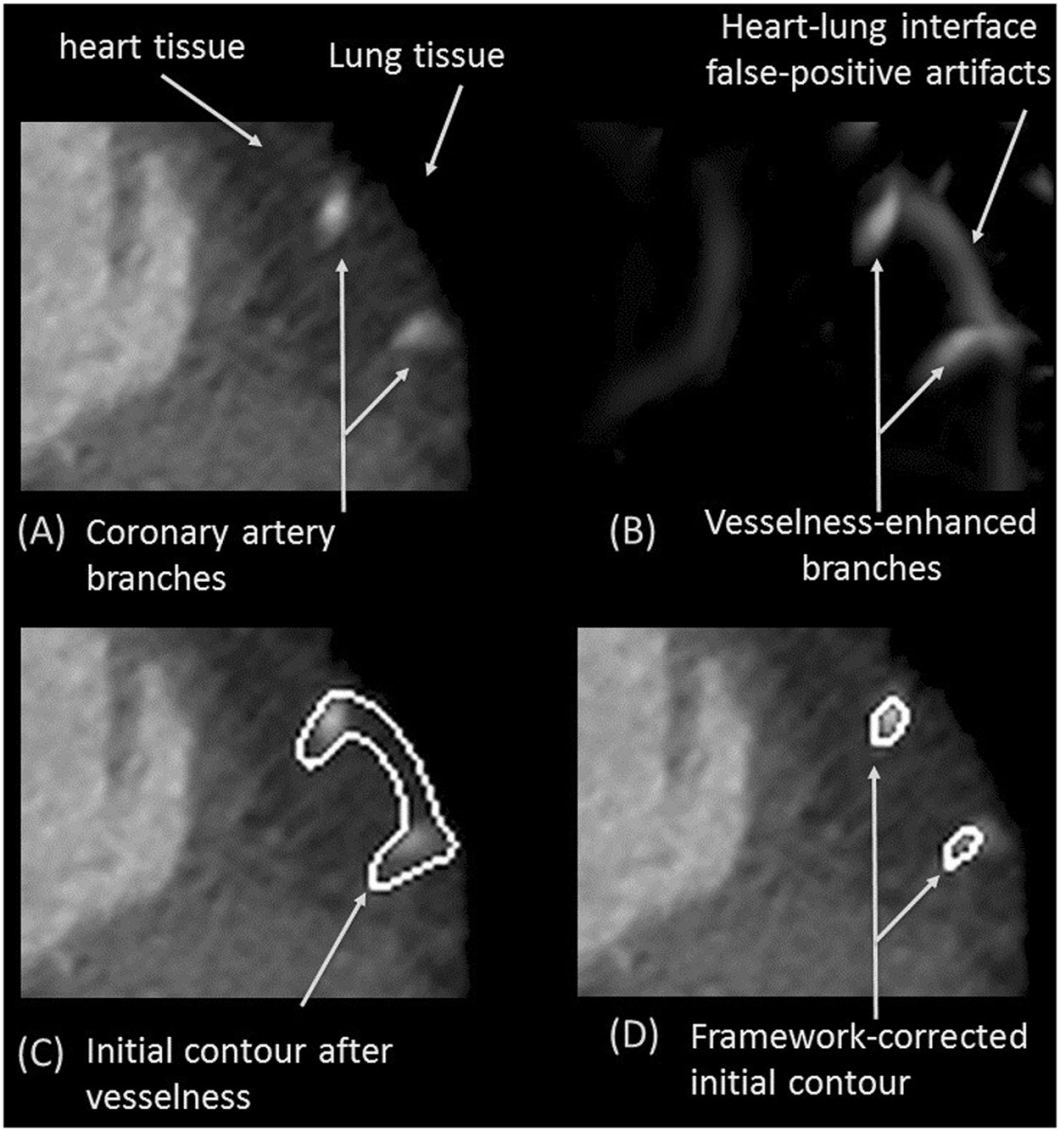
Improvement in initial contours; avoiding false vesselness artifacts. Coronary branches in the original CCTA (**A**) and artefactual shapes are enhanced with vesselness (**B**). Level set segmentation based on vesselness mistakenly includes these false structures (**C**). Proposed framework correctly identifies the vessel from the other objects (**D**).

The first new approach is for extracting coronary lumen. In other vessel segmentation problems, it was sufficient to initialize the level set algorithm with seed points or with a rough inexact initial contour. However, that approach is not sufficient for coronary CTA and is prone to early failure and pre-mature termination where vessels at certain locations can be distorted due to motion artifacts, severe stenosis, or blooming artifacts due to large calcification. In the proposed framework, the initial contouring step involved filtering out vessel-like background structures and generating a more precise robust initial contour. These tasks were accomplished by combining vesselness and region growing as was shown in Figs 1 and 7.

The second new approach presented in this work is for coronary outer wall segmentation in which the final level set lumen contour is utilized to generate the initial contour for the outer vessel boundaries. The resulting initial outer contour is quite close to the vessel outer boundary, including inside it all of the potential plaques and excluding surrounding fat. This initial outer contour was then used by the second level set routine to extract the final outer vessel wall.

While there are benchmarks for centerline calculation, lumen segmentation, and stenosis detection^18,30^, there is no benchmark yet for vessel wall and plaque segmentation. To validate our segmentation, a blinded comparison between the three expert observers’ manual delineation and framework segmentation versus the expert observers’ consensus demonstrated a successful framework. This novel framework had the least volume MSE, the least wall relative error, and was better than at least one observer in the other metrics. Moreover, when the study senior radiologist with the closest performance to the consensus was asked to fix the framework segmentation to correct what was viewed as clearly a wrong segmentation, the mean difference in DICE, precision, and sensitivity was always within 10% in each plaque category and got smaller as plaques got larger. These results clearly demonstrate the frameworks successful performance and suggest that the framework segmentation can be a significant step in CCTA plaque analysis workflow to accelerate radiologist’s performance and reduce the inter-observer variability.

**Figure 8:**
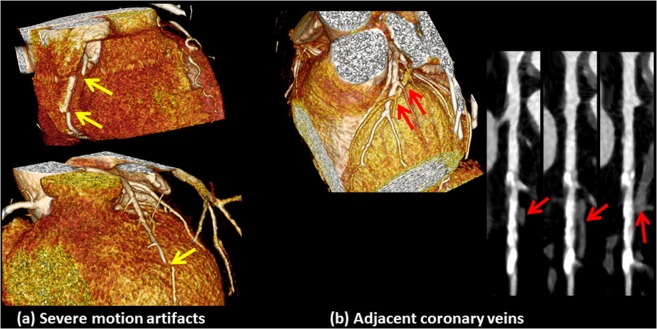
Examples of cases in which the framework will fail due to (**a**) severe motion artifacts, in which parts of the coronary artery are broken and discontinuous (yellow arrows), and (**b**) coronary veins that are in close proximity to or in contact with the arteries and are misclassified as plaques (red arrows).

Successful segmentation requires diagnostic quality images of the coronary tree. The performance of the framework may be compromised under certain situation, which can be grouped into three categories. The first category includes sites with motion-induced blurring artifacts, blooming artifacts from calcified plaque, or lack of perceived fat tissue between the artery and the myocardium. Neither a radiologist nor the algorithm can determine with certainty the extent of plaques in these circumstances. Yet, the presented framework will still preserve the continuity of the segmentation through these sites of coronary vasculature and proceed with segmenting the rest of the tree. The second category of situation includes sites with severe motion artifacts that cause discontinuity of the artery, as shown in Fig. 8 left, or the segments of the arteries that tangent the coronary veins, as shown in Fig. 8 right. Coronary veins have a HU range similar to the wall and plaque tissues and will be misclassified as plaques unless manually guided. A radiologist can correctly interpret this category with higher degree of confidence than the framework as it requires a higher order spatial recognition than what the framework currently has.

In conclusion, automatic CCTA coronary wall segmentation with high performance compared to radiologist’s manual delineation is feasible regardless of the degree of stenosis. This framework provides a robust working platform for accelerated, objective, and reproducible atherosclerotic plaque characterization and quantification beyond subjective assessment of stenosis and can be potentially applicable for monitoring patients at CHD risk and the response to therapy.

## Materials and Methods

The details of framework’s modules are presented in this section. We, first, present the module for the initial inner and outer contour calculation. Next, we describe the segmentation and visualization modules. Specific implementation steps including subroutines, libraries, and toolkits of the proposed framework are listed in the appendix.

### Initial contours

Level set techniques have been commonly used for solving 3D vessel segmentation problems because of their robustness and insensitivity to small intra-region intensity variations and their ability to overcome a suboptimal initialization^31^. Thus, our proposed framework uses level sets to identify the final segmentation of both the lumen and the vessel.

**Figure 9:**
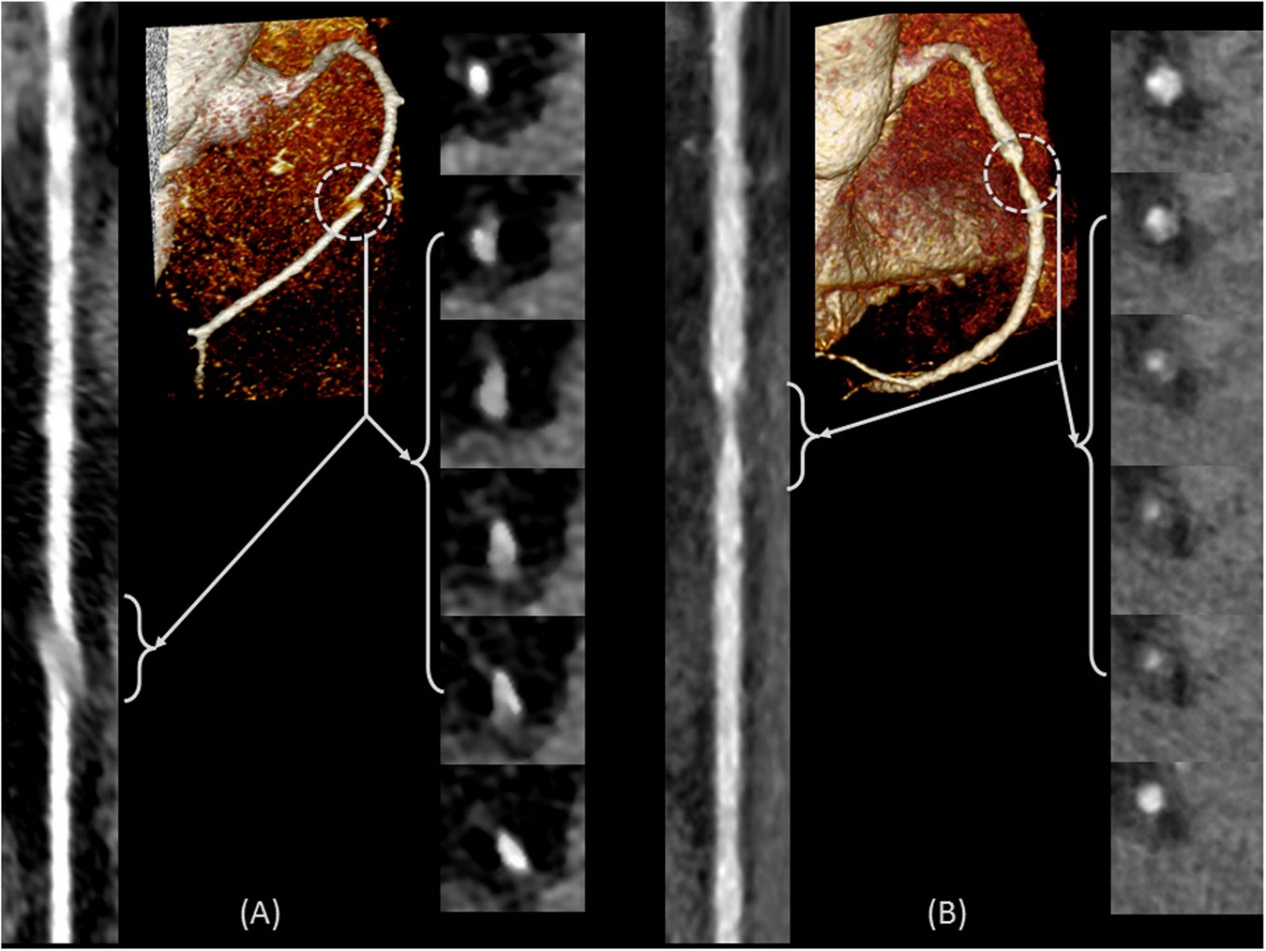
Examples of changes in HU due to motion artifacts (**A**) and severe stenosis (**B**), which will lead to level set failure and early termination if used without the proposed initial contouring.

However, level sets alone will likely provide an inaccurate, and possibly completely erroneous contour of the coronary lumen in situations with substantial artifacts or poor image acquisition quality. The small caliber of the coronary arteries which is comparable in size to the imaging resolution and the large underlying motion, along with the lumen narrowing due to stenosis, can cause large variations within lumen brightness in Hounsfield units (HU). These intra-lumen variations are comparable to inter-vessel HU intensity variations from lumen to surrounding tissues. Severe stenosis and motion artifacts examples are shown in Fig. 9 to demonstrate cases in which lumen HU drop substantially, in such cases a level set lumen segmentation will terminate abruptly at the sites of severe motion artifacts or severe stenosis; even though the segments with artifacts are followed by other segments of diagnostic value. We avoid this early termination by preceding the level set segmentation with an automatically generated initial contour near to the lumen boundary, which will serve as a guide to the level set segmentation and will prevent it from early termination in artifactual segments (Fig. 1).

In the ideal case, Gaussian functions can model the change of lumen intensity profile across the vessel while a low change in gray levels is expected in the longitudinal direction along the vessel. As a result, a zero-crossing of the first derivative associated with a peak in the second derivative are expected across the vessel lumen. Based on this principle, many techniques proposed second derivative analysis^32–34^ to enhance and/or detect the vessels.

Utilizing the Hessian matrix to perform second derivative analysis provides a rotational and translation invariant tool for pre-processing and enhancing tube-like shapes. However, it is not scale invariant, which is important for dealing with multiple vessel sizes. Applying the second derivative after convoluting the image with a set of Gaussian functions with different standard deviations can resolve this issue.

**Figure 10:**
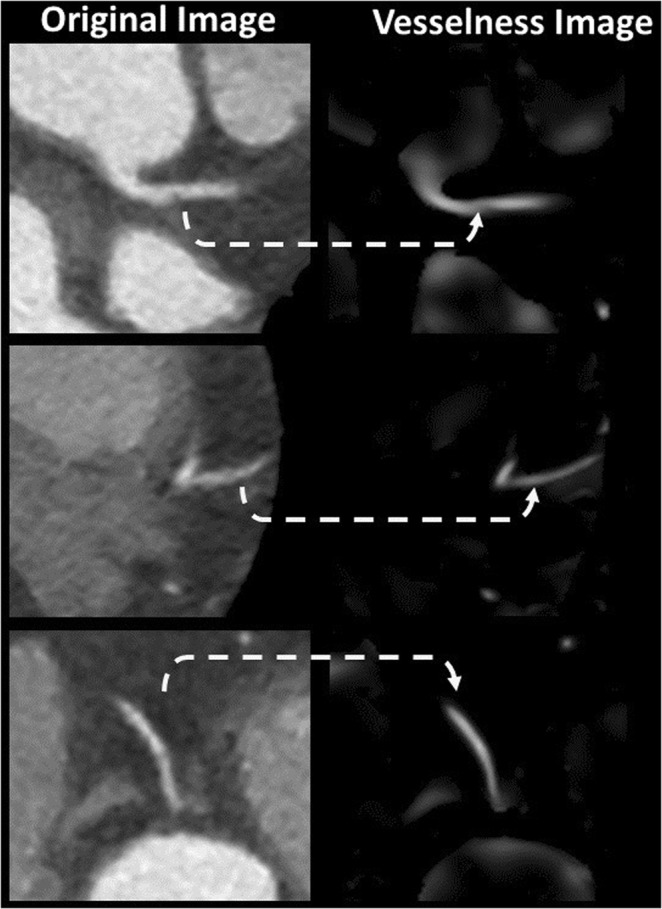
Examples of original CCTA data (left) after the application of the vesselness filter (right). Tubular and sharp-curvature structures are enhanced, which includes vessels and vessel-like structures.

In this work, Frangi’s vesselness function^34^ is used to enhance the appearance of the coronary arteries in the input 3D CTA dataset. An example of the outcome of the vesselness filter is shown in Fig. 10 where all tube-like and sharp-curvature structures are enhanced compared to the background and other objects.

The vesselness filter often enhances non-vessel structures as well. Particularly when coronary arteries pass near the borders of the heart-lung interface, these borders are erroneously enhanced by the vesselness filter creating false positive branches. Therefore, the output of this module is not ready to be used alone as an initial contour for segmentation with level sets. Figure 7(A) shows one of these cases where two branches of left coronary artery are near the heart-lung interface, the vesselness outcome is shown in Fig. 7(B). The figure shows the false positive branch that joins the two coronary branches.

To avoid such false vesselness-enhanced structures and to generate a more faithful initial contour, we propose adding a region-growing module to the framework as shown in Fig. 1. Region-growing is applied to both the original CTA image and the vesselness image independently. The two binary outputs are intersected thereafter. Applying region-growing on the vesselness image extracts the targeted coronary arteries, RCA, LCA, LCX and the false branches previously enhanced in the vesselness module. Applying region-growing on the original image, the output is a connected region that contains the coronary arteries, the aorta, and all other pixels connected to any of these arteries and have HU in the same range as blood HU. The intersection of both results provides a region representing the coronary arteries only without the false branches or the aorta and other connected pixels. Figure 7 demonstrates an example of this step. The vesselness filter applied to the original data generates false structures (Fig. 7(B)), which results in the wrong borders for the initial region (Fig. 7(C)). Correct borders for the region are identified from the proposed framework (Fig. 7(D)).

The final lumen inner wall contour is then calculated from the initial lumen contour using a level set method. The segmented lumen is used to calculate the initial vessel outer wall contour. Previous literature indicates large plaques causing severe stenosis ≥40% have a thickness of 4.38 ± 0.71 mm^22^. Consequently, we extend the segmented lumen by 5.5 mm using restricted morphological dilation. The restricted dilation excludes the surrounding fat regions, identified as pixels with negative HU, from the vessel initial contour. The resulting shape is considered the vessel initial outer contour that encloses the vessel and the plaques. In the segmentation module, this initial outer contour will shrink inward toward the actual outer boundaries of the vessel wall as described in the next section.

### Segmentation

Segmentation of the lumen and vessel, which correspond to the inner and outer final contours, respectively, is performed using a level set method. We evolve two level sets in opposite directions; one from inside the lumen toward the true lumen boundary followed by the other level set starting from outside the vessel inward to the vessel boundary. The method takes the initial lumen and vessel contours as input and the feature image as guidance as described in the previous section (Fig. 1). The feature image is calculated by applying the sigmoid function on the gradient image.

**Figure 11:**
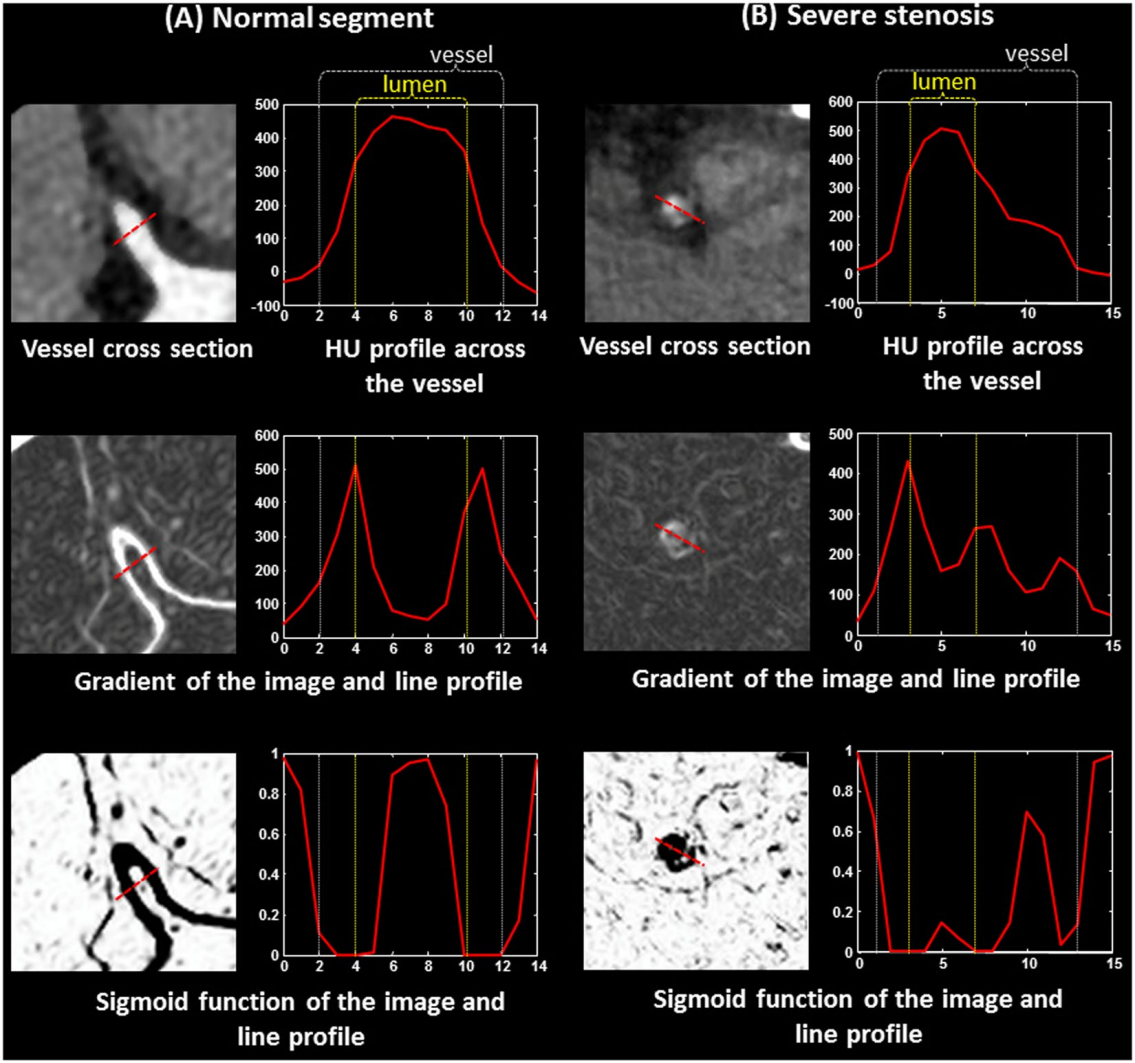
Examples of the calculation of the feature image of the same subject used for the lumen and vessel level set segmentation module at a normal segment (**A**) and at a stenotic segment (**B**).

Figure 11 shows examples of the line profiles of the HU image (top), the gradient image (middle), and the feature image in Fig. 11 (bottom) across the lumen and wall of (Fig. 11(A)) a healthy vessel segment and (Fig. 11(B)) a segment with a non-calcified plaque of the same subject. The change in the HU across the healthy vessel wall is relatively symmetric, smooth, and steep while the change through the plaque is slower and irregular. In both cases, points with maximum gradients cannot be used to reliably represent either the inner or the outer boundaries. First, the points with maximum HU gradient are of substantially lower HU intensity values than the center of the lumen. Second, in healthy cases, only one gradient peak exists that neither corresponds to the lumen inner wall nor the vessel outer wall. As shown in Fig. 11 (top), a more accurate representation of the lumen and vessels are the inner and the outer shoulders of the HU profile, respectively.

### Visualization

The second part of the framework is the visualization of results. Once the wall is segmented, curved multiplanar reformation (CMPR) technique is used to straighten the original 3D dataset, and the segmented lumen and wall^30^ using the lumen centerline as a guideline.

Vessel centerline is calculated in two steps. In the first step, the Marching Cube algorithm is employed to extract a zero-level set that is used to generate the 3D mesh of the inner surface of the vessel wall. Next, the centerline is obtained from the 3D mesh using the Voronoi diagram^35^. The resulting centerline is analyzed using Frenet–Serret formulas^36^ that describe the geometry of the centerline at each point along it by three orthogonal unit vectors: tangent, normal, and binormal unit vectors. These vectors guide the CMPR of the original 3D input image, the inner level set image, and the outer level set image. An example of the centerline and CMPR output for the left coronary arteries is shown in Fig. 2 demonstrating the lumen centerlines, different coronary segments, and their representations in the straightened vessels.

After calculating CMPR for the inner and outer level set images, Marching Cube is applied to the 3D inner and outer segmented walls to extract the inner and outer contours which are displayed as an overlay on the reformatted input image as shown in Fig. 4. Finally, the vessel wall average thickness is calculated at every cross section and plotted along the longitudinal the straightened artery.

Further details about implementation subroutine and software libraries used in the construction of the framework can be found in the appendix.

## Supplementary information

Appendix Implementation Routines and Libraries
