# Supplementary material for: Automatic Coronary Wall and Atherosclerotic Plaque Segmentation from 3D Coronary CT Angiography
Source: Sci Rep. 2019 Jan 10;9:47. doi: 10.1038/s41598-018-37168-4 (PMC6328572; doi:10.1038/s41598-018-37168-4)
Supplement: Supplementary file 1 — Appendix Implementation Routines and Libraries [file 41598_2018_37168_MOESM1_ESM.pdf]

# Automatic Coronary Wall and Atherosclerotic Plaque Segmentation from 3D Coronary CT Angiography

Ahmed M. Ghanem[1], Ahmed H. Hamimi[1], Jatin R. Matta[1], Aaron Carass[2], Reham M. Elgarf[1], Ahmed M. Gharib[1], Khaled Z. Abd-Elmoniem[1]

**Author information**:

[1]The Biomedical and Metabolic Imaging Branch, National Institute of Diabetes, Digestive, and Kidney Diseases, National Institutes of Health, Bethesda, MD, USA

[2]The Image Analysis and Communications Laboratory, Department of Electrical and Computer Engineering, Johns Hopkins University, Baltimore, MD, USA

***Correspondence:**

Khaled Z. Abd-Elmoniem, PhD, MHS

National Institutes of Health

Bldg.10, Rm.3-5340, MSC 1263

10 Center Drive, Bethesda, MD 20892, USA

Tel.: +1 301 451 8982, Fax: +1 301 496 9933

E-mail: abdelmoniemkz@mail.nih.gov

## APPENDIX: IMPLEMENTATION

The framework was developed as a plugin of Osirix® (ver. 6.5.1, 64-bit) plugin on Mac OS (MacBook Pro, Intel Core i7, 2.8 GHz, Radeon R9 M370X 2048 MB) using Objective-C, C++, and three open source toolkits; the Insight Segmentation and Registration Tool Kit (ITK ver. 4.7, https://itk.org/ITK/resources/software.html) for general image enhancement and processing routines, the Visualization Tool Kit (VTK ver. 5.9, https://www.vtk.org/download/) for marching cube and 3D mesh generation, and the Vascular Modeling Tool Kit (VMTK ver. 1.0, http://www.vmtk.org/download/) for implementing vesselness, centerline, and CMPR calculations. Table A lists the framework components, algorithms, used toolkits and implantation classes.

The modules and block diagram of the proposed framework were previously demonstrated in Figure 1 and further explained in expanded forms in the appendix Figures A for the segmentation modules and visualization modules in Figure B below. The implementation details of every modules are further described in the following paragraphs.

**Data preparation module:** In this preparation step, input image format was converted to VTK and ITK images formats from Digital Imaging and Communications in Medicine (DICOM) image format. VTK and VMTK toolkits use and generate the same image format, vtk::vtkImageData, while ITK uses its own data format, itk::Image. Two additional image format converters, vtk::vtkImageData to itk::Image and vice versa, were used during the transformation of the images among the toolkits to assure compatibility. To reduce the execution time, only the region of interest (ROI) was converted and processed by the framework. The 3D ROIs were automatically generated around the seed points. The radiologist was able to manually adapt the ROIs if needed.

**Lumen initial contour module:** As shown in Figure A, generating the lumen initial contour was performed using vesselness, region growing, and image intersection algorithms. The VMTK was utilized for implementing the Frangi's vesselness while ITK performs the connected threshold region growing algorithm and image intersection as listed in Table A.

**Feature image module:** The gradient image was calculated using gradient magnitude with smoothing algorithm then the sigmoid function was applied. The generated feature image was then

used for both lumen and vessel segmentation. This module was implemented using only ITK as listed in Table A.

**Level Set segmentation module:** The Geodesic level set algorithm was used for segmenting both lumen and vessel. The module inputs were the initial contour and the feature image. The vessel initial contour was calculated using the segmented lumen as described in the next paragraph. In lumen segmentation, the level set evolution parameters were set to extend the lumen initial contour to the lumen boundary. While in vessel segmentation, the parameters were set to shrink the initial contour toward to the vessel boundary. The level set segmentation was performed using VMTK.

**Vessel initial contour module:** The vessel initial contour was calculated by dilating the segmented lumen up to 5.5 mm to insure including the vessel and the plaques within the initial contour. In the segmented lumen image, the pixels within the lumen have negative values while the surrounding pixels have positive values. As a result, we dilated the lumen by applying morphological erosion. To get more accurate initial contour, the surrounding fat regions, identified as pixels with negative HU in the original image, were excluded.

**3D mesh generation module:** This module was used during the visualization part for generating 3D meshes from the segmented images. The Marching Cube algorithm was applied on the segmented images to extract a zero-level set that was used by triangulation algorithm to generate an initial 3D mesh. The final 3D mesh was acquired by utilizing Laplacian smoothing algorithm. The implementation of this module was performed using VTK as listed in Table A and Figure B left side.

**Centerline extraction module:** VMTK was utilized to obtain the lumen centerline from the lumen 3D mesh by the Voronoi diagram algorithm. The VMTK implementation of Frenet–Serret formula was then applied to analyze the geometry of the centerline which was described at each point along the centerline by three orthogonal unit vectors: tangent, normal, and binormal unit vectors. (Figure B right side)

**Curved multi-planer reformation (CMPR) module:** The performing of the CMPR was guided by the lumen centerline. Frenet–Serret formulas' unit vectors were used by CMPR algorithm to calculate the orthogonal and tangent plans at each centerline's point. The CMPR module was applied on original input image, the lumen image, and the vessel segmentation image. The VMTK was used for the

implementation of this module (Figure B bottom).

Table A. Implementation submodules, toolkits, algorithms, and classes.

| Module | Toolkit | Algorithm | Class |
|---|---|---|---|
| Vesselness | VMTK | Frangi Vesselness | vtkvmtkVesselnessMeasureImageFilter |
| Region Growing | ITK | Connected Threshold | ConnectedThresholdImageFilter |
| Intersection | ITK | Minimum | MinimumImageFilter |
| Gradient | ITK | Gradient Magnitude with Smoothing | GradientMagnitudeRecursiveGaussianImageFilter |
| Sigmoid | ITK | Sigmoid Function | SigmoidImageFilter |
| level set | VMTK | Geodesic level set | vtkvmtkGeodesicActiveContourLevelSetImageFilter |
| Morphological Operator | ITK | Grayscale Erosion | GrayscaleErodeImageFilter |
| Marching Cube | VTK | Marching Cube | vtkMarchingCubes |
| 3D Mesh Generator | VTK | Triangulation and Laplacian smoothing | vtkTriangleFilter<br>vtkSmoothPolyDataFilter |
| Centerline Generator | VMTK | Voronoi diagram and Frenet–Serret formulas | vtkvmtkPolyDataCenterlines<br>vtkvmtkCenterlineGeometry |
| CMPR | VMTK | CMPR | vtkvmtkCurvedMPRImageFilter |

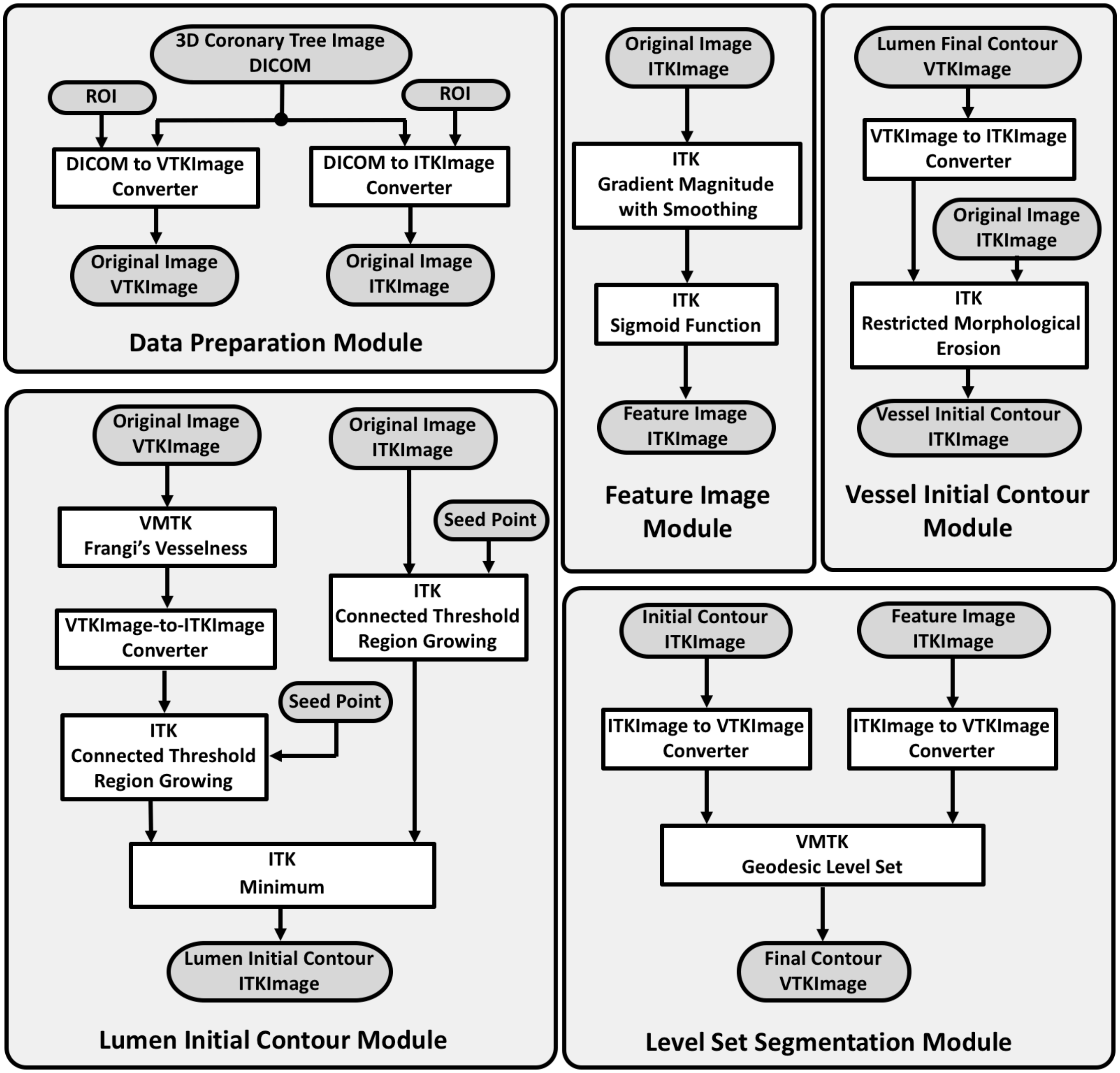


Figure A. Segmentation block modules

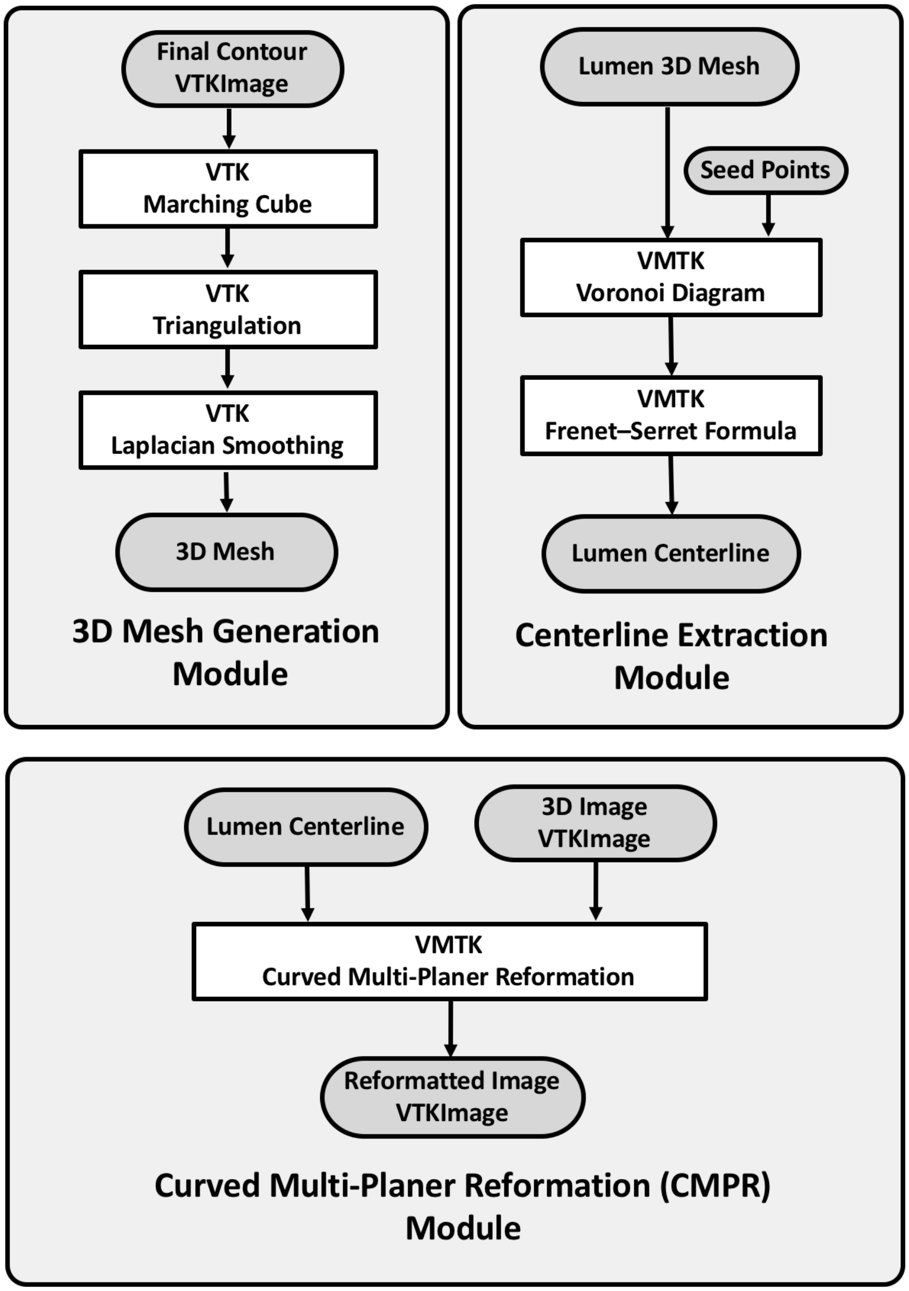


Figure B. Visualization block modules
